# Scoping review for the SAGES EAES joint collaborative on sustainability in surgical practice

**DOI:** 10.1007/s00464-024-11141-x

**Published:** 2024-08-22

**Authors:** Bright Huo, M. M. M. Eussen, Stefania Marconi, Shaneeta M. Johnson, Nader Francis, Wendelyn M. Oslock, Nana Marfo, Oleksii Potapov, Ricardo J. Bello, Robert B. Lim, Jonathan Vandeberg, Ryan P. Hall, Adnan Alseidi M. D. EdM, Manuel Sanchez-Casalongue, Yewande R. Alimi, Andrea Pietrabissa, Alberto Arezzo, Maximos Frountzas, Vittoria Bellato, Paul Barach, Miran Rems, Sheetal Nijihawan, Tejas S. Sathe, Benjamin Miller, Sarah Samreen, Jimmy Chung, N. D. Bouvy, Patricia Sylla

**Affiliations:** 1 Department of General Surgery, McMaster University, Ontario, CA USA; 2 Department of Surgery, Maastricht University Medical Center, Maastricht, The Netherlands; 3 NUTRIM School of Nutrition and Translational Research in Metabolism, Maastricht University, Maastricht, The Netherlands; 4 Department of Civil Engineering and Architecture, University of Pavia, Pavia, Italy; 5 IRCCS Policlinico San Matteo Foundation, Pavia, Italy; 6 Department of Surgery, Morehouse School of Medicine, 720 Westview Drive, Atlanta, GA 30310 USA; 7 Griffin Institute, London, UK; 8 Department of Surgery, University of Alabama Birmingham, Birmingham, AL USA; 9 Department of Quality, Birmingham Veterans Affairs Medical Center, Birmingham, AL USA; 10 Department of General Surgery, College of Medicine, University of Rzeszow, Rzeszow, Poland; 11 Ross University School of Medicine, Miramar, FL USA; 12 Department of Surgery, Medical College of Wisconsin, Milwaukee, NC USA; 13 Department of Surgery, Atrium Carolinas Medical Center, Wake Forest University, Charlotte, USA; 14 Department of Surgery, Mayo Clinic Florida, Jacksonville, FL USA; 15 Department of Surgery, Tufts Medical Center, Boston, USA; 16 Department of Surgery, University of California San Francisco, San Francisco, USA; 17 Department of Surgery, Clinica San Camilo, Buenos Aires, Argentina; 18 Department of Surgery, Medstar Georgetown University Hospital, Washington, DC USA; 19 Department of General Surgery, University of Pavia, Pavia, Italy; 20 Department of Surgical Sciences, University of Turin, Turin, Italy; 21 First Propaedeutic Department of Surgery, Hippocration General Hospital, National and Kapodistrian University of Athens, Athens, Greece; 22 Department of Minimally Invasive Surgery, University Hospital of Rome Tor Vergata, Rome, Italy; 23 Thomas Jefferson University School of Medicine, Philadelphia, USA; 24 Department of General Surgery, Imperial College London, London, UK; 25 Department of General and Abdominal Surgery, General Hospital Jesenice, Jesenice, Slovenia; 26 Department of Surgery, Sharon Regional Medical Center, Sharon, PA USA; 27 Cleveland Clinic Foundation, Cleveland, OH USA; 28 Division of Minimally Invasive Surgery, University of Texas Medical Branch, Galveston, TX USA; 29 Adventus Health Partners, Cincinnati, OH USA; 30 Division of Colon and Rectal Surgery, Mount Sinai Health System, New York, NY USA

**Keywords:** Sustainability, Minimally invasive surgery, Climate change, Greenhouse gas emissions, Recycling, Surgical waste

## Abstract

**Background:**

Surgical care in the operating room (OR) contributes one-third of the greenhouse gas (GHG) emissions in healthcare. The European Association of Endoscopic Surgery (EAES) and the Society of American Gastrointestinal and Endoscopic Surgeons (SAGES) initiated a joint Task Force to promote sustainability within minimally invasive gastrointestinal surgery.

**Methods:**

A scoping review was conducted by searching MEDLINE via Ovid, Embase via Elsevier, Cochrane Central Register of Controlled Trials, and Scopus on August 25th, 2023 to identify articles reporting on the impact of gastrointestinal surgical care on the environment. The objectives were to establish the terminology, outcome measures, and scope associated with sustainable surgical practice. Quantitative data were summarized using descriptive statistics.

**Results:**

We screened 22,439 articles to identify 85 articles relevant to anesthesia, general surgical practice, and gastrointestinal surgery. There were 58/85 (68.2%) cohort studies and 12/85 (14.1%) Life Cycle Assessment (LCA) studies. The most commonly measured outcomes were kilograms of carbon dioxide equivalents (kg CO_2_eq), cost of resource consumption in US dollars or euros, surgical waste in kg, water consumption in liters, and energy consumption in kilowatt-hours. Surgical waste production and the use of anesthetic gases were among the largest contributors to the climate impact of surgical practice. Educational initiatives to educate surgical staff on the climate impact of surgery, recycling programs, and strategies to restrict the use of noxious anesthetic gases had the highest impact in reducing the carbon footprint of surgical care. Establishing green teams with multidisciplinary champions is an effective strategy to initiate a sustainability program in gastrointestinal surgery.

**Conclusion:**

This review establishes standard terminology and outcome measures used to define the environmental footprint of surgical practices. Impactful initiatives to achieve sustainability in surgical practice will require education and multidisciplinary collaborations among key stakeholders including surgeons, researchers, operating room staff, hospital managers, industry partners, and policymakers.

**Supplementary Information:**

The online version contains supplementary material available at 10.1007/s00464-024-11141-x.

Climate change is recognized as the greatest public health threat of the twenty-first century [1]. Climate change is associated with an increase in infectious diseases, respiratory, neurological and cardiovascular complications, as well as mortality [2]. It has also been reported to adversely affect mental health, pregnancy, nutrition [2]. Healthcare is the second-leading contributor to waste in the United States [3], and is responsible for 10% of the nation’s greenhouse gas (GHG) emissions [4]. Surgery is responsible for up to one-third of waste in healthcare [5]. Surgical care in Canada, the United Kingdom, and the United States emits 9.7 million tons of carbon dioxide equivalents (CO_2_eq) per year, with the equivalent climate impact of 2 million motor vehicles [6]. Operating rooms (OR) are the most resource-intensive area of the hospital due to the use of consumables, anesthetic gases, sterilization processes, and significantly high energy requirements [7, 8]. Gastrointestinal surgery produces the third-highest output of solid waste per case relative to other specialties [5]. The rapid adoption of minimally invasive surgery (MIS) has accelerated the reliance on single use equipment, waste production, and energy consumption. The clinical benefits of MIS must be weighed against its relative contribution to the OR carbon footprint [5, 9].

Hospitals and health sector companies are systematically decarbonizing the healthcare supply chains in line with the US & European Union’s pledge to cut GHG emissions in half by 2030[10, 11] Although initiatives targeted at reducing carbon emissions in the operating room are anticipated to be among the most impactful within healthcare, organizational leadership in such efforts has been slow and disjointed [12, 13]. Healthcare organizations have had limited success in engaging perioperative leadership. One obstacle to developing sustainable surgical practices is a lack of knowledge among perioperative teams about the relative contribution of operative elements to the carbon footprint [7, 14]. The limited understanding among healthcare providers, lack of standardized protocols, and absence of evidence-based guidelines, has limited efforts to implement green initiatives, especially due to the uncertainty of overall impact of the carbon footprint.

The EAES (European Association of Endoscopic Surgery) and SAGES (Society of American Gastrointestinal and Endoscopic Surgeons) created a Sustainable Surgical Practices (SSP) Joint Task Force in response to this global environmental challenge (https://eaes.eu/sustainability-in-surgical-practice/; SUSTAINABILITY IN SURGICAL PRACTICE—SAGES). The aim is to develop actionable recommendations to inform members of the best practices in mitigating climate risk in surgical practice, specifically in MIS. The SSP Task Force embarked on a scoping review of all relevant publications on the topic of sustainability measures, practice, and initiatives in the field of gastrointestinal surgery.

## Materials and methods

The EAES and SAGES conducted a scoping review in alignment with methodological guidance from the JBI Scoping Review Methodology Group [15]. We followed the reporting standards for the Preferred Reporting Items for Systematic Review and Meta-Analysis Extension for Scoping Reviews (PRISMA-ScR) according to our protocol (supplementary appendix 1) [16]. The review aimed to address the following research questions:

- What is the fundamental, core terminology in sustainability research relevant for surgeons and researchers?
- Are there evidence-based outcome measures used in sustainability research?
- What is the scope of sustainability relevant to gastrointestinal surgical practice?
- What is the climate impact of surgical practice, including:
- All general surgical procedures performed in the operating room.
- Anesthesia practice (e.g., choice of anesthetic).
- Any practices in the operating room that may impact the environment.
- What evidence exists to support the practical implementation of green surgery initiatives to achieve sustainable surgical practice?

### Literature search & inclusion criteria

**Fig. 1:**
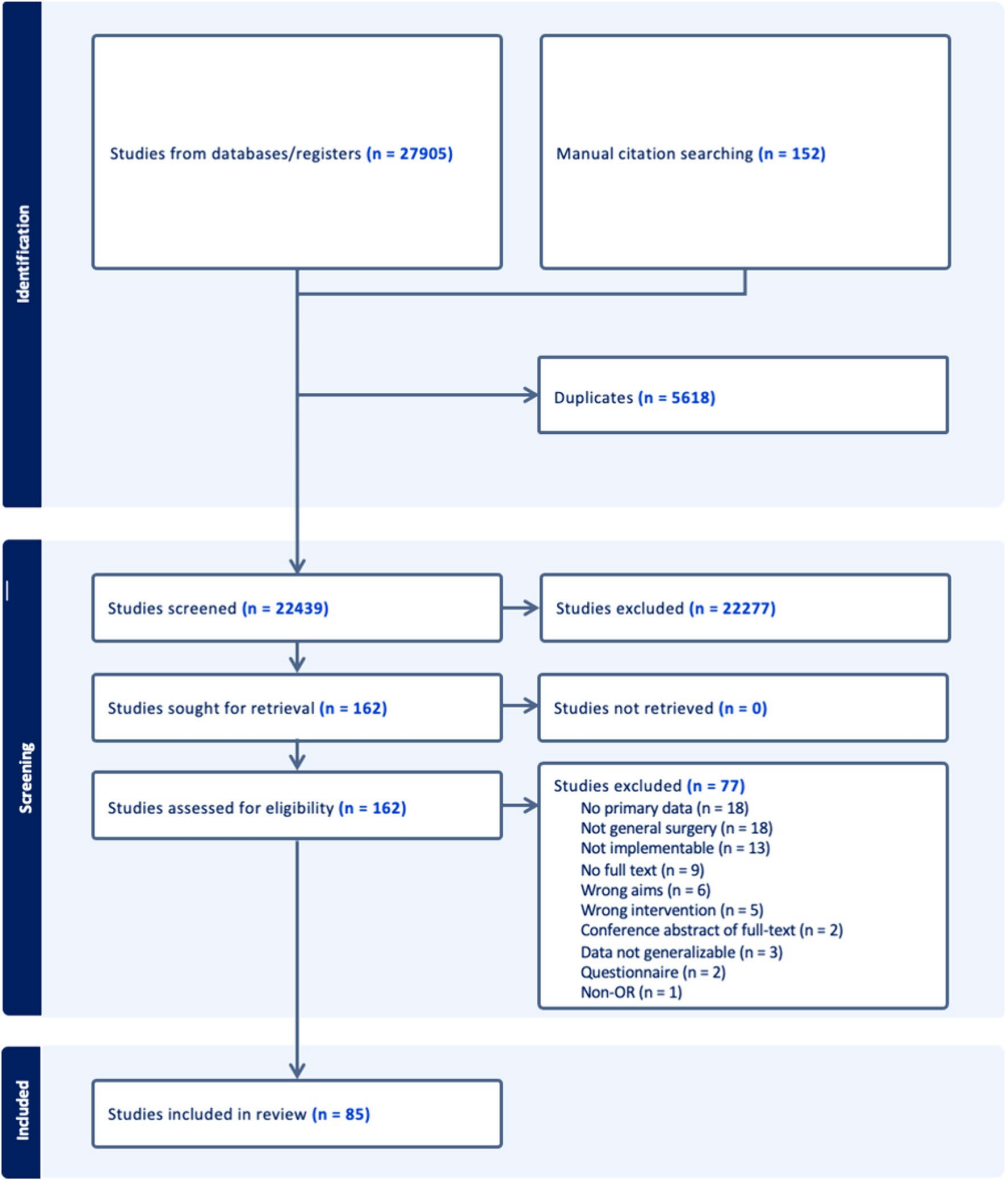
Literature search overview

A comprehensive search strategy was developed with the help of an academic health sciences librarian with expertise in scoping reviews (Fig. 1). MEDLINE via Ovid, Embase via Elsevier, Cochrane Central Register of Controlled Trials, and Scopus were queried from inception to August 25th, 2023 to identify key articles of interest (Table 1). Backward and forward manual citation searching were conducted on all included articles to identify additional articles of interest. Search syntax is available in supplementary appendix 2.

**Table 1:** Inclusion & exclusion criteria

	Inclusion criteria	Exclusion criteria
Design	RCTs, prospective studies, observational studies Life-cycle assessments Conference proceedings	^*^Non-primary literature Systematic reviews (SR), meta-analyses (MA) Standards of practice Case reports, questionnaires, narrative reviews, commentaries, editorials, letter to the editor, opinion articles
Population	Studies assessing topics of sustainability in: ^1^Gastrointestinal surgical practice Adult and pediatric surgery ^2^General practice in the operating room	Animal studies Veterinary studies Studies assessing sustainability topics notrelated to ^1^gastrointestinal surgical practice Studies assessing endoscopic ^3^procedures Studies assessing infrared-guided procedures or interventions
Aims	Studies assessing the ^4^climate impact of gastrointestinal surgical practice Studies assessing practically implementable ^5^interventions or initiatives to promote sustainable surgical practice	Studies assessing basic science topics (non-clinical) Studies assessing interventions or initiatives in surgery with novel, innovative technology, or equipment Studies evaluating clinical outcomes of interventions or initiatives in surgery
	Studies evaluating cost of interventions or initiatives in surgery	Studies describing global surgery initiatives
		Studies assessing telehealth or telemedicine

^1^Gastrointestinal surgery defined as below:

All procedures performed in the operating room such as laparoscopic cholecystectomy, totally extraperitoneal hernia repair (TEP).

Includes open and minimally invasive (laparoscopic & robotic) surgery

^2^General practice in the operating room:

Anesthesia practice (e.g., choice of anesthetic)

Air treatment practices

Any practices in the operating room that may impact the environment

^3^Endoscopic procedures: colonoscopy, esophagogastroduodenoscopy, laryngoscopy, cystoscopy, etc.

^4^Climate impact:

Climate change (CO_2_eq)

### Study selection & data extraction

Records identified by the systematic literature search were uploaded to Covidence for the screening phase. A review team was led by a review coordinator with experience in systematic and scoping review methodology. Literature screening was performed in two rounds; first by title and abstract and second by full-text. Each article was screened by a minimum of two reviewers. Prior to the first round of screening, a pilot screening round was conducted with a sample of 25 abstracts. After abstracts were screened independently, the review team met to calibrate reviewer selections. Next, the first round of screening by title and abstract was conducted using the inclusion criteria outlined in Table 1. Prior to entering the second round of screening, pilot screening was completed using five full-text articles. An additional meeting was held to calibrate reviewer selections. Finally, the second round of full-text screening was conducted. Throughout both rounds of screening, reviewers met to resolve discrepancies and when necessary, a senior author was available to reconcile disagreements.

Included articles from both rounds of screening entered data extraction. Data extraction from included studies was conducted independently, while each article was extracted by two team members. In cases of discrepancies, resolution was achieved through consensus. For any unresolved discrepancies, a senior author with content expertise was consulted to achieve resolution.

### Data analysis

Quantitative data were summarized using descriptive statistics. Baseline characteristics of included articles were summarized using counts and percentages, including practice setting, study design, the reporting of educational/behavior change programs, and the use of core outcome measures applied in included studies for analysis. Descriptive analysis was supported by graphical representations in diagrammatic form when applicable. A narrative summary was developed to accompany the findings.

### Scope of sustainability in surgical practice

Studies available in the literature spanned all three scopes delineated in the Greenhouse Gas (GHG) Protocol (https://ghgprotocol.org/) [17, 18]. Measurements of the environmental impact of costly and environmentally harmful anesthetic gases and the multiple interventions to reduce anesthetic gas emissions fall under Scope 1. Initiatives to decrease energy consumption in the OR, whether by implementing occupancy-based LED lighting, anesthetic gas scavenging, or Heating, Ventilation, & Air Conditioning (HVAC) systems, fall under Scope 2 of the GHG Protocol. Additionally, studies focusing on waste reduction and the carbon footprint of the supply chain would be categorized into Scope 3. Water consumption, which is not captured by the GHG Protocol, is equally an important scope in the climate impact of surgical care. Studies were classified according to study design as cohort studies, randomized controlled trials (RCTs), computational studies, correspondence articles with primary data, and LCA studies.

### Concepts, terminology, and definitions

**Fig. 2:**
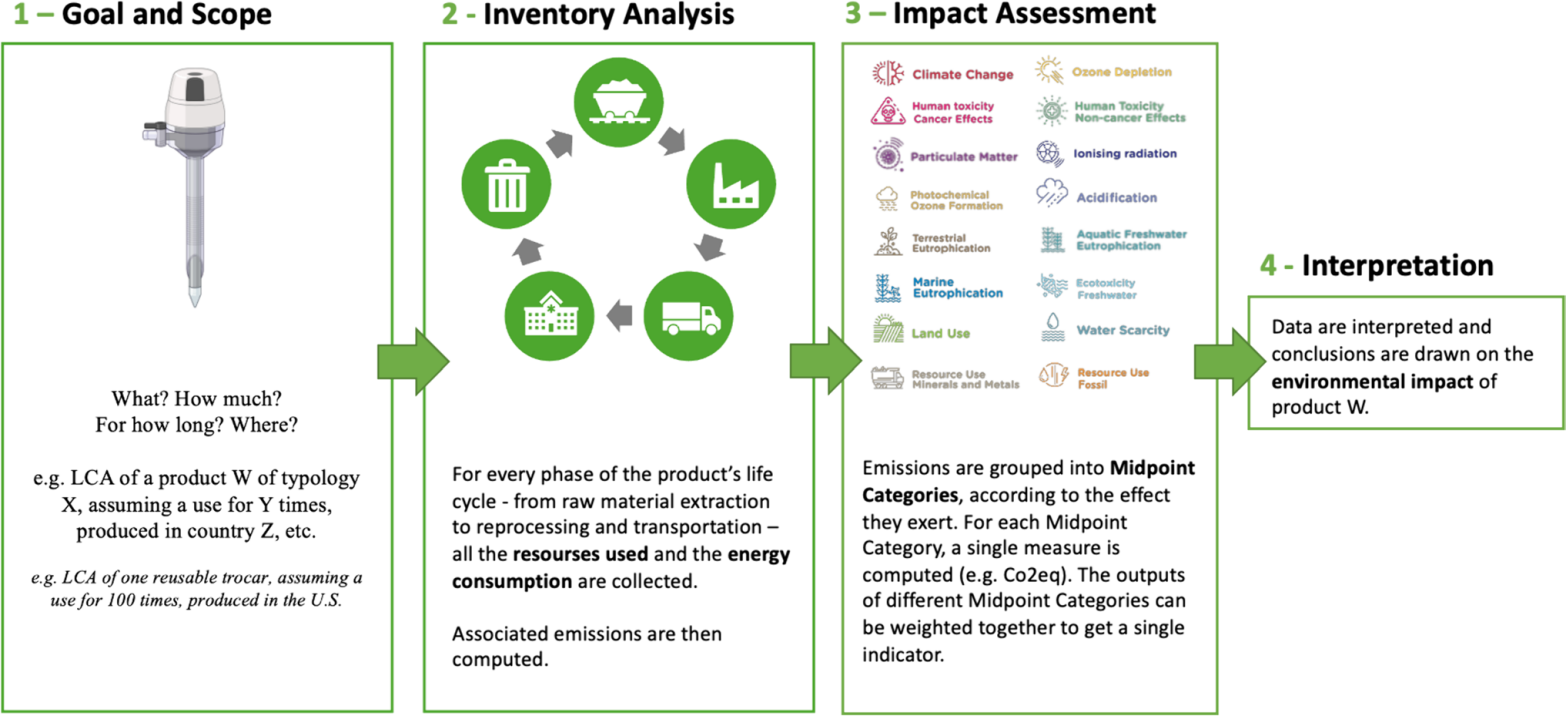
Overview of life-cycle assessment

Key concepts, terminology, and definitions applied in the assessment and measurement of sustainability in surgical practice are summarized in Table 2. LCA is defined as a systematic approach to evaluate and quantify the environmental impacts associated with a product, process, or service, examining every stage in its life cycle. LCAs are widely recognized as the benchmark for measuring environmental impact [19]. ISO 14040 and 14,044 are international standards which provide an important framework for LCAs. Unique outcomes can be measured in LCAs including terrestrial, freshwater, and marine ecotoxicity, human carcinogenic toxicity, land use, ionizing radiation, and marine eutrophication (Fig. 2) [20].

**Table 2:** Glossary of core terminology for sustainability in gastrointestinal surgical practice

Terms	Definitions
Principles	
Circular economy	A model of economy that involves activities that are restorative or regenerative by design and aims for the elimination of waste through the superior design of materials, products, and systems
Climate change	Shifts in weather and climate patterns that occur over long periods of time, acknowledging that the current warming temperature is caused primarily by human activity
Decarbonize	The act of reducing the amount of GHG emissions associated with a process or product, with the goal of being net neutral
Green	A colloquial term to refer to initiatives, products or practices that have environmental benefits such as reduced use of environmental resources
Greenhouse gas (GHG)	A gas (primarily CO_2_ but including CH_4_, N_2_O, and others) that absorbs, traps, and re-emits heat and radiant energy back into the earth's atmosphere
Planetary health	An emerging concept that prioritizes solutions that simultaneously benefit human health and advance environmental sustainability
Sustainability	Meeting the needs of the present without compromising the ability of future generations to meet their own (UN Brundtland Commission)
Study Designs	
Life Cycle Assessment (LCA)	Life cycle assessments are a rigorous methodology for studying the environmental impact of a product or process. LCAs consider both the upstream and disposal of products to capture all inputs and outputs. Outcomes measured LCAs include a comprehensive range of outcome measures. While time intensive, these are the gold standard for sustainability studies
Outcome Measures	
Carbon dioxide equivalents (CO_2_eq)	A measure of the carbon dioxide required to generate a corresponding amount of climate impact, which allows for comparison. It is an outcome measure used to compare the emissions from various greenhouse gases based on their global-warming potential (GWP), by converting amounts of other gases to the equivalent amount of CO_2_ with the same global warming potential. CO_2_eq are expressed as unit of mass. The International System of Units (SI) unit of mass is the kilogram (consider that multiples—as tons—often applies)
Cost	An economic measure of resource consumption most commonly in euros, US dollars, or some other currency
Ecosystem quality (PDF*m^2^*year or species.year)	A measure of the effect on biodiversity caused by climate change, marine acidification, land use, toxic substances (freshwater ecotoxicity), substances that cause terrestrial and aquatic acidification, freshwater and marine eutrophication. It can be expressed as potentially disappeared fraction (PDF)* of species over a certain area over a certain time (PDF.m2.yr) or as species over year (species.yr).[104]
Energy (kWh, MJ)	A measure of the amount of energy used to deliver surgical care. The International System of Units (SI) unit of energy is the joule (megajoules—MJ—typically applies), while the non-SI unit is the energy delivered by one kilowatt of power for one hour (kWh)
Human health (DALY/person/year)	A measure of the impact of climate change on human health in disability-adjusted-life-years (DALY). One DALY represents the loss of the equivalent of one year of full health. The unit commonly used to express Human Health is the number of DALYs per person per year (DALY/person/yr).[105]
Waste (kg)	A measure of the amount of waste generated to deliver surgical care, typically express as a mass. Less commonly reported in L
Water (L)	A measure of the amount of water used to deliver surgical care, typically expressed as a volume. Less commonly reported as m^3^ or kg

**Fig. 3.**
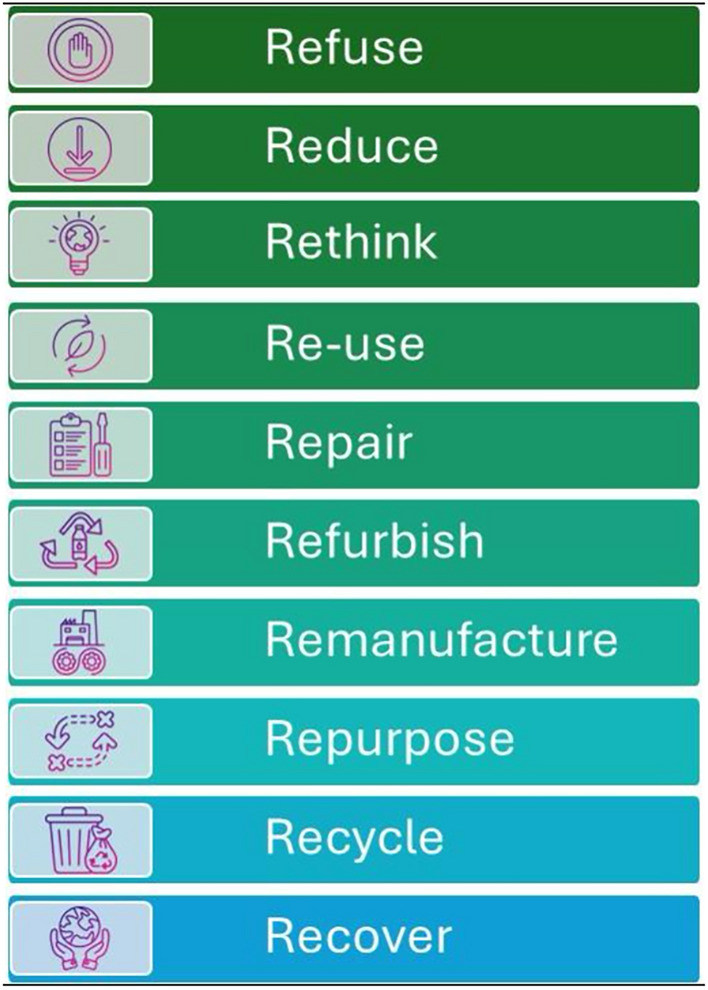
10R model of circular economy

Another key concept with a strong framework in sustainability research is the 10R model of the circular economy. [21] This approach prioritizes strategies based on their potential to minimize emissions. These strategies are ranked by their effectiveness and include the need to refuse, reduce, rethink, reuse, repair, refurbish, remanufacture, repurpose, recycle, and recover (Fig. 3) [21]. By adopting these practices, organizations can transition toward a more sustainable and circular approach to design and production. The goal is to minimize waste, conserve resources, and create products that contribute positively to the environment and society. We used the 10R model to organize and present the findings of the scoping review.

## Results

A total of 22,439 articles were screened to yield 85 relevant papers (Fig. 1). Studies were conducted in the settings of anesthesia (*n* = 30/85, 35.3%), general surgical practice (*n* = 35/85, 41.2%), and gastrointestinal surgery (*n* = 21/85, 24.7%). There were 51/85 (60.0%) prospective studies, 7/85 (8.2%) retrospective studies, 9/85 (10.6%) correspondence articles, and 12/85 (14.1%) LCA studies (Table 3). No studies applied the 10R model. With respect to scope, there were 17/85 (20.0%) articles related to Scope 1 (anesthetic gas), 4/85 (4.7%) articles to Scope 2 (energy), 53/85 (62.4%) to Scope 3 (waste), and 4/85 (4.7%) articles to water consumption (Table 3).

**Table 3:** Characteristics of studies evaluating sustainability in the operating room for gastrointestinal surgery, general surgical practice, and anesthesia

Author	Country	Study design	Single vs multisite	Aim	Scope	Surgical procedure
Gastrointestinal surgery						
Adler, 2005	Germany	Case study	NR	Reusable vs disposable	3*	Laparoscopic cholecystectomy
Agarwal, 2010	India	Prospective	Single	Energy	2	Laparoscopic cholecystectomy
Billings, 2017	USA	Prospective	–	Waste—impact	3	Elective colectomy
Bischofberger, 2023	Switzerland	Prospective	Single	Anastomotic leaks	3	–
Blankush, 2021	USA	LCA	–	Open vs robotic	3	Ventral hernia repair
Boag, 2022	UK	Prospective	Multisite	Leaner equipment	3	Laparoscopic appendectomy
Boberg, 2022	Sweden	LCA	–	Reusable vs disposable	3	Laparoscopic cholecystectomy
Brady, 2017	USA	Prospective/Retrospective	–	Reusable vs disposable	3	Laparoscopic colectomy
Caycedo-Marulanda, 2020	Canada	Correspondence with primary data	Multisite	Anesthetic gas	1	taTME
Colak, 2004	Turkey	RCT	–	Reusable vs disposable	3	Laparoscopic cholecystectomy
Dettenkofer, 1999	Germany	LCA | –	–	Waste	3	–
Ford, 2022	UK	Prospective	Single	Leaner equipment	3	Laparoscopic appendectomy
Gough, 2022	UK	Prospective		Leaner equipment	3	Laparoscopic cholecystectomy
Graham, 2019	USA	Retrospective	–	Waste—impact	3	Laparoscopic appendectomy
Labib, 2023	UK	Prospective	Multisite	Leaner equipment	3	Laparoscopic appendectomy
Park, 2021	USA	Prospective	–	Education—surgeon report card	3	Multiple
Petterwood, 2009	Australia	Prospective	–	Water	-	–
Rizan, 2022	UK	LCA	Single	Reusable vs disposable	3	Laparoscopic cholecystectomy
Robb, 2022	UK	Prospective	–	Anesthetic gas	1	Inguinal hernia repair
Sullivan, 2023	USA	Prospective	–	Waste—impact	3	Multiple pediatric
Vacharathit, 2021	USA	Correspondence				
With primary data	–	Education—Fellowship program	1,2,3	–		
General surgical practice						
Burguburu, 2022	France	LCA	Multisite	Reusable vs disposable	3	–
Cannings, 2022	UK	Correspondence				
With primary data		Water—scrubbing	-	Multiple		
Chasseigne, 2018	France	Prospective	Multisite	Waste—impact	3	Multiple
Conrardy, 2010	USA	Correspondence				
With primary data	Single	Reusable vs disposable	3	Multiple		
Cunningham, 2023	USA	Retrospective/ Prospective	Single	Leaner equipment	3	Multiple
Friedericy, 2022	The Netherlands	LCA	Multisite	Waste—recycling	3	–
Gilliam, 2008	UK	Correspondence				
With primary data	Single	MIS	3	Multiple laparoscopic general surgery		
Ibbotson, 2013	Germany	LCA	Single	Reusable vs disposable	3	–
Jehle, 2008	UK	Retrospective/Prospective	Single	Water—scrubbing	–	Multiple
MacNeill, 2017	Canada/USA/UK	LCA	Multisite	Waste—impact	3	Multiple
Martin, 2022	France	Correspondence				
With primary data	Multisite	Waste- recycling	3	Multiple		
Martin, 2017	United States	Prospective	Single	Waste—recycling	3	Multiple
McKendrick, 2017	United Kingdom	Prospective	Single	Waste—recycling	3	Multiple
Moreno, 2019	Spain	Computational	Single	Waste—impact	2	–
Ramos, 2023	Denmark	Prospective	Single	Energy—air recycling	3	Multiple
Rizan, 2022	UK	Retrospective	Single	Reusable vs disposable	3	Multiple
Rouvire, 2022	France	LCA	Multisite	Waste—recycling/disposal	3	Multiple
Sadler, 2017	UK	Prospective	Single	Waste—impact	3	Multiple
Singleton, 2019	UK	Prospective	Single	Reusable vs disposable	3	–
Sinha, 2023	UK	Correspondence				
With primary data	Single	Waste—recycling	3	Simple skin excision		
Somner, 2008	UK	Prospective	Single	Waste—impact	3	–
Tieszen, 1992	USA	Retrospective	–	Water—scrubbing	–	Multiple
Van Straten, 2021	Netherlands	Prospective	Multisite	Waste—recycling	3	–
Vozzola, 2020	USA	LCA	Single	Reusable vs disposable	3	–
Wormer, 2013	USA	Prospective	Multisite	Reusable vs disposable	3	–
Wyssusek, 2016	Australia	Prospective	Single	Waste—impact, recycling	3	–
Wyssusek, 2020	Australia	Prospective	Single	Waste—impact, recycling	3	–
Amariglio, 2021	Italy	Prospective	Single	Waste—impact	3	Multiple
Dohmen, 2023	Germany	Prospective	Single	Waste—impact	3	Multiple
Mhlaba, 2015	USA	Prospective	Single	Reusable vs disposable	3	Multiple
Nast, 2019	USA	Prospective	Single	Reusable vs disposable	3	Multiple urology
Power, 2012	USA	Retrospective	Single	Leaner equipment	3	Multiple
Rizan, 2022	UK	LCA	Single	MIS	3	–
Rizan, 2023	UK	Prospective	Multisite	Reusable vs disposable	3	Multiple
Anesthesia						
Alexander, 2018	Canada	Correspondence				
With primary data	Multisite	Anesthetic gas	1	–		
Atcheson, 2016	USA	Prospective	Single	Anesthetic gas	1	Multiple
Baloi, 2018	UK	Prospective	Multisite	Waste—impact	3	Multiple
Benness, 2021	Australia	Prospective	Single	Anesthetic gas	1	Multiple
Boyle, 2018	UK	Prospective	Multisite	Anesthetic gas	1	Multiple
Carter, 2019	UK	Prospective	Single	Anesthetic gas	1	–
Caycedo-Marulanda, 2022	Canada	Correspondence with primary data	Single	Anesthetic gas	1	–
Chambrin, 2023	France	Retrospective	Single	Anesthetic gas	1	Multiple
Connolly, 2019	Australia	Prospective	Single	Waste—recycling	3	Multiple
Davies, 2023	Australia	Prospective	Multisite	Waste—impact, recycling	3	Multiple
Fraifeld, 2021	USA	Prospective	Single	Waste– impact, recycling	3	Multiple
Glenski, 2020	USA	Prospective	Multisite	Anesthetic gas	1	Multiple pediatric
Hansen, 2023	USA	Prospective	Single	Anesthetic gas	1	Multiple pediatric
Hickman, 2021	UK	Prospective	Multisite	Energy	2	Multiple
Hubbard, 2017	USA	Prospective	Single	Anesthetic gas	1	Multiple
Ito, 2021	UK	Prospective	Single	Anesthetic gas	1	Multiple
Kaniyil, 2017	India	Prospective	Single	Waste—impact	3	Multiple
Langbein, 1999	Germany	Laboratory	Single	Waste—impact	3	–
Livingston, 2019	Scotland	Prospective	Single	Waste—recycling	3	Multiple
Mankes, 2012	USA	Prospective	Single	Waste—impact	3	Multiple
McGain, 2009	Australia	Prospective	Single	Waste—impact	3	Multiple
McGain, 2010	Australia	LCA	Single	Waste—impact	3	Multiple
McGain, 2014	Australia	Prospective	Single	Waste—impact	3	Multiple
Nesaratnam, 2018	UK	Prospective	Single	Waste—impact	3	Multiple
Pierce, 2014	UK	Prospective	Single	Energy	2	Multiple
Ryan, 2010	US	Laboratory	Single	Anesthetic gas	1	–
Sherman, 2012	USA	LCA	Single	Anesthetic gas	1	–
Sulbaek Andersen, 2010	USA	Prospective	Single	Anesthetic gas	1	Multiple
Wyssusek, 2022	Australia	Retrospective	Single	Anesthetic gas	1	Multiple
Zuegge, 2019	USA	Retrospective	Single	Anesthetic gas	1	Multiple

*Three Scopes defined in the GHG Protocol (https://ghgprotocol.org/) [17, 18]

Scope 1: Measurements of the environmental impact of costly and environmentally harmful anesthetic gases and the multiple interventions to reduce anesthetic gas emissions

Scope 2: Initiatives to decrease energy consumption in the OR, whether by implementing occupancy-based LED lighting, anesthetic gas scavenging, or HVAC systems

Scope 3: Additionally, studies focusing on waste reduction and the carbon footprint of the supply chain

### Outcome measures

Outcomes evaluated in the included studies demonstrated substantial heterogeneity. The most commonly studied outcome measure was CO_2_eq in 39/85 (45.9%) studies, with most studies measuring kilograms (kg) CO_2_eq. The cost of resource consumption was the second most commonly studied outcome across 32/85 (37.6%) studies in US dollars, euros, or some other form of currency. Surgical waste was measured in 28/85 (32.9%) studies and were typically reported in units of mass (kg) or volume in liters (L). Water consumption was evaluated in 11/85 (12.9%) articles in liters (L) or less commonly in kg. Energy consumption was assessed in 7/85 (8.2%) studies in kilowatt-hours (kWh), or less commonly in megajoules (MJ). The impact on human health was measured in disability-adjusted life-years (DALYs) in 3/85 (3.5%) articles, and ecosystem damage (PDFm^2^/yr) in 2/85 (2.4%) studies. Key outcomes studied in LCAs include the climate change impact (kg of CO2eq), ecosystem damage (PDFm2yr),

### Climate impact

**Fig. 4:**
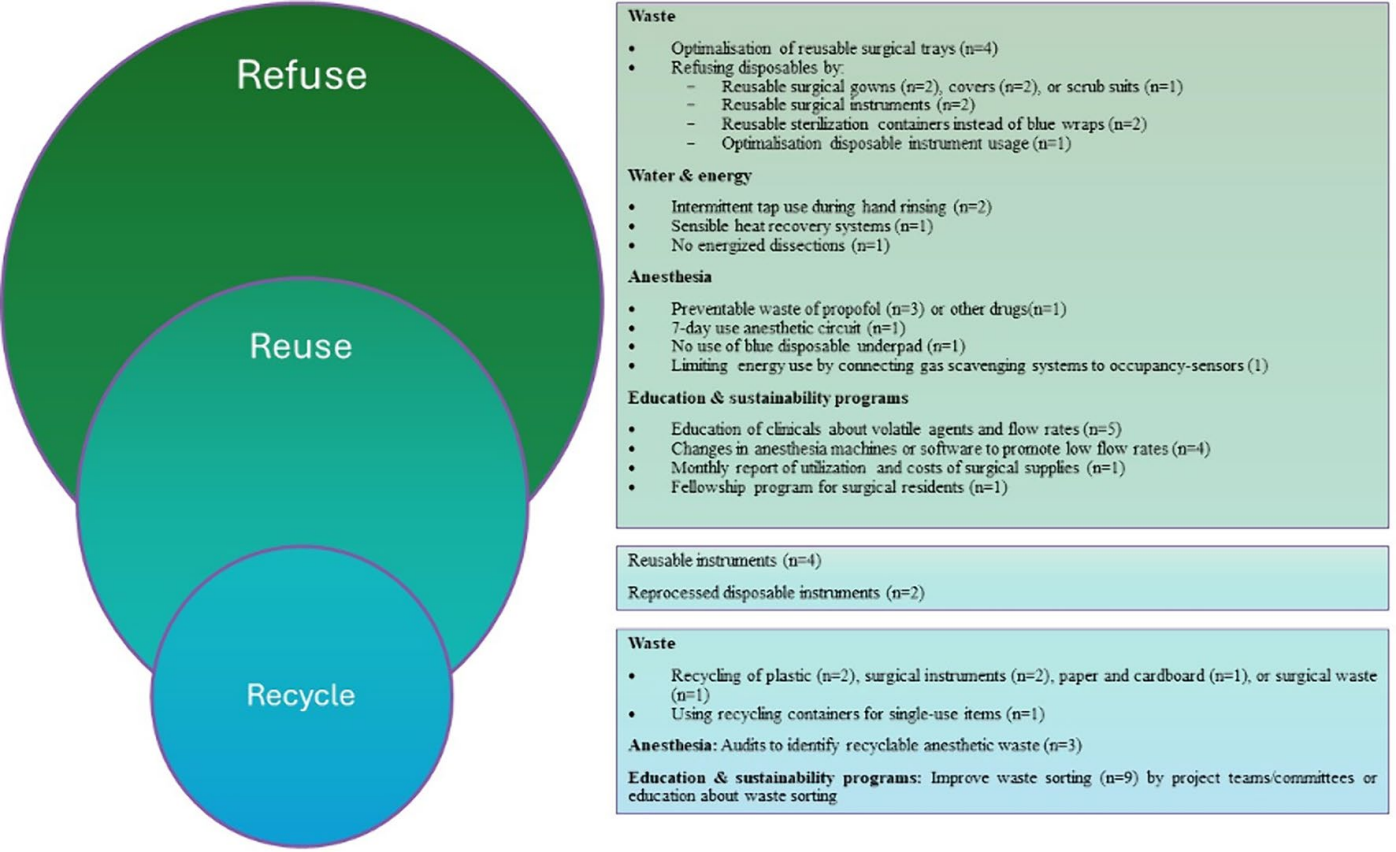
Study findings aligned with the 10R model

Surgical care exerts up to 5,187,936 kg of CO2eq annually from one academic institution, alone [6]. Some contributors to climate impact are undesired, but to some extent uncontrollable. For example, anastomotic leaks are associated with an average climate, water and waste impact per patient of 1303kgCO_2_eq, 1803m^3^ of water and 123 kg waste, respectively, including diagnosis, inpatient stay in addition to treatment and monitoring [22]. Thus, it is helpful to focus on contributors to climate change that are within the control of surgical teams. Studies included in this review did not apply the 10R framework, but their findings can be re-conceptualized using portions of this framework including refuse, reuse, and recycle (Fig. 4).

#### Refuse

Waste: It is estimated that $368 of waste is generated per case across general surgical procedures due to unnecessary equipment use of sutures, staplers reload, and other items [23]. This amounts to a total preventable annual cost of $18,410 across 50 common general surgery cases [23]. Chasseigne et al. reported that wasted supplies represent 20.1% of the total cost allocated to surgical supplies [24]. For common procedures such as appendectomy, it is estimated that 75% of equipment-related consumables could be reduced [25]. Up to 59% of surgical instrument trays may be unused, leading to unnecessary water and energy uses for sterilization. [26]

Water, Energy, & MIS: There is interest in the use of alcohol-based scrubs to mitigate water use. Canning and colleagues showed that 1,606,692L of water would be saved with alcohol-based rather than soap and water-based scrubbing, equating to 2.2 tons of carbon saved annually [27]. Similarly, Jehle et al. showed that over 15,500 procedures performed in a 1-year period, 931,938L of water would be saved with alcohol-based agents [28].

Anesthesia: Anesthetic gases have been known to significantly impact the environment since 1999.[29] Desflurane exerts the greatest impact on global warming compared to isoflurane or sevoflurane [30], even after accounting for resource extraction and manufacturing of anesthetic drugs, transport to health care facilities, clinical use, and disposal or emission to the environment [31]. Caycedo-Marulanda and colleagues showed a lower impact on CO_2_eq with sevoflurane (8.4 kg) compared to desflurane (408.6 kg) for transanal total mesorectal excision (taTME) surgery [32]. Given the higher costs and environmental impact of desflurane, some hospitals have chosen to stop using this agent altogether. Caycedo-Marulanda et al. found that when their facility implemented a desflurane-free strategy in their operating rooms there was a significant reduction in CO_2_eq [33]. Hansen et al. combined the removal of desflurane from the formulary with avoiding nitrous oxide, decreasing flow rates, and applying educational initiatives. These practices reduced GHG emissions by 87% and CO_2_eq from volatile anesthetics > 50% [34].

#### Rethink

Waste: Considering alternative approaches to surgical care can mitigate the impact of surgery on the environment. It has been shown that the use of clips for appendectomies produces 10.2 g of waste per case compared to 975.1 g per case with staplers, resulting in equivalent efficacy and cost savings of $286.33 per case in 2019 [35]. Additionally, sterilization processes may be modified with the use of rigid sterilization containers to reduce the carbon footprint by 85% compared to disposable blue wraps, with an ecological advantage after 98 of 5,000 use cycles. Moreover, rigid sterilization container use resulted in 84.5% less environmental impact in terms of eco-costs, with an ecological advantage already occurring after 67 out of 5000 use cycles [36].

MIS: In addition to waste, MIS exerts additional impact on the overall surgical footprint. Blankush et al. performed a LCA that demonstrated that robotic approaches consume 143% more energy than open approaches for ventral hernia repair, equating to an additional 1.4 barrel of oil equivalents per procedure, resulting in 144% (609 kg CO_2_eq) more GHG emissions. Robotic ventral hernia repair resulted in 18% more solid waste per case; with downstream environmental impact causing an incremental loss of 1.9 × 10^–3^ DALYs per case [37]. In laparoscopic surgery, Gillam reported that climate impact from CO_2_ use alone in 2006 was minimal [38]. However, Power et al. reviewed over two million laparoscopic cases performed in the USA in 2009, showing that laparoscopic procedures produced 355,924 tons of CO_2_/year when accounting for the transportation, capture, and compression of CO_2_. [39] Notably, length of stay was not captured in these studies, and it has been established that MIS reduces the length of stay, the rate of complications, and healthcare costs compared to open surgery [40]. The impact of these factors on the climate impact of MIS has yet to be elucidated.

Anesthesia: Robb et al. reported a saving of 10.2 kgCO_2_e/case by using local anesthetic instead of general anesthesia for inguinal hernias [41]. Ensuring appropriate triage of the urgency of cases may also ameliorate the impact of surgical care, as an analysis of four anesthetic gases showed that the highest CO_2_kg/hr was recorded for emergency (17.6), followed by elective cases (7.7) due to excessive gas flow rates [42].

#### Reduce

Waste: Many initiatives aimed to reduce surgical waste. Boag et al. downsized laparoscopic appendectomy instrument trays from 119 to 49 items, saving 7.48kgCO_2_eq and £25.1 per procedure [43]. Leaning appendectomy trays to reduce consumables can save a net £34,423- £219,452 and 512kgCO_2_eq -3.02 tons CO_2_eq per year over a predicted seven-year life span of instruments [25, 44]. Despite an investment needed to lean equipment trays, the investment of £19,731 was recuperated within six months [25]. Elsewhere, Cunningham and colleagues removed 46 items across 113 pediatric procedures which, saved $27,503 in surgical equipment acquisition costs and prevented > 6,000 tons of waste annually [45]. Similarly, authors reduced instruments trays for orchiopexy and inguinal hernia repairs from 57 to 35 instruments, which saved $3,489.42 annually [46].

In contrast, Sinha and colleagues analyzed the waste generated by full drape coverage, fully gowned and gloved surgeon versus a pragmatic draping policy with single fenestrated drape and only sterile gloves, for minor surgeries as simple skin incisions. The full approach produced 596 g of plastic waste per patient (£14.62 per patient) while pragmatic draping policy in 120 g of plastic waste per patient (£8.53/ per patient) [47].

Water & Energy: With respect to mitigating the use of water, Petterwood et al. compared two scrub techniques: continuous tap flow versus intermittent tap use during hand rinsing. The latter resulted in a 71% water savings (15.5L reduced to 4.5L) [48]. Similarly, Somner and colleagues reported that having the tap on for only one as opposed to two minutes reduced the use of water by 5.7L per surgical scrub [49].

Mitigating the use of energy can also improve the climate impact of surgery. Authors have shown that installing a sensible heat recovery system in the operating room recirculates 50% of the airflow in the theater, reducing energy demands by 44.3%, equating to 49, 261.9 kWh/year, saving €7,389.29 annually [50]. Conversely, Agarwal et al. reported a reduction from 57 to 39 L CO_2_ per laparoscopic cholecystectomy without energized dissection [51].

Anesthesia: In addition to anesthetic gases, other medications used in anesthesia are costly, resource intensive to produce, and contribute to environmental contamination. Atcheson et al. looked at the preventable anesthetic waste in 543 separate surgical cases and found that the estimated yearly cost of preventable anesthetic drug waste was $185,250 [52]. Studies by Baloi and Butt, Kaniyil et al., and Mankes found that the highest volume of drug waste is propofol [53–55]. This finding led to a change in formulary at one hospital with 50 mL and 100 mL bottles of propofol exchanged for 20 mL bottles, with a resultant decrease in propofol waste from 29 mL/day/bin to 3 mL/day/bin [55].

McGain et al. (2014) evaluated the difference in microbial contamination with different frequency of anesthetic circuit changes in the setting of single use airway filters. They found that there was no difference in proportion of circuit contamination if changed every 24 h (57/105), 48 h (43/100) or 7 days (46/100). This resulted in $4,846 savings per year (for 6 ORs) as well as decreased sterilizer loads saving 2760 kWH per year and 48,000 L of water [56]. Furthermore, Pierce et al. attempted to mitigate energy use by using meters of outlets for machines and in-use energy estimates for the anesthetic gas scavenging system. The total energy use per day for anesthesia was 28kWH per theater, with the majority (18kWH) of this energy coming from the anesthetic gas scavenging system [57]. Hickman et al. found that 14 of 29 anesthetic gas scavenging systems were on inappropriately, generating 26,980kWH of unnecessary energy use. By connecting the scavenging system to existing occupancy-sensors used for the HVAC system, they reduced the time the system was running when the operating room was unoccupied, preventing the generation of 14.3 tons of CO_2_ across the 29 OR system in a single year [58].

Education/Sustainability Programs: Initiatives to educate surgical staff on the climate impact of surgery have been implemented. Park et al. applied a monthly surgeon report card detailing the utilization and cost of disposable and reusable surgical supplies on cost and waste reduction for pediatric laparoscopic procedures, showcasing a reduction of 43% of costs (from $631 to $235 median supply cost per case). This also reduced the use of disposable trocars by 56% and of disposable harmonics and staplers by 33% [59]. Vacharathit et al. reported the 5-year results of a fellowship program for surgical residents in healthcare sustainability, primarily focused on greening the OR which resulted in 116,865 gallons of water saved per year with education on water waste, scrub-less surgical solutions and motion activated faucets; 1 million pounds of plastics diverted from landfill, regulated medical waste reduced by 26 tons per month with waste segregation education and additional recycling sorting assistance; and $53,075 savings and reduction in 717 metric tons of CO_2_ equivalents per year with automated lighting linked to OR occupancy [60].

Zuegge et al. educated anesthesia providers on volatile agent choices and flow rates, decreasing emissions by 64% (in terms of CO_2_eq). This was driven by decreased use of desflurane, with associated cost savings of $25,000/month [61]. Benness et al. designed a series of educational interventions to decrease use of desflurane, which accounted for 80% of the volatile carbon footprint in their department. Their education interventions resulted in a 58% reduction in the use of desflurane over the course of 9 months, which equated to > $46,000 in cost savings and avoidance of 360 ton CO_2_eq [62]. Wyssusek et al. implemented several educational interventions regarding inhaled anesthetics and reduced combined sevoflurane and desflurane usage costs and emissions by 58.33% and 87.88%, respectively, over the 6 years following implementation [63]. Furthermore, Davies et al. provided education on anesthetic gas selection to an Australasian network focused on sustainable anesthesia practices through an initiative titled Operation Clean Up. This reduced the volume of desflurane used from 1.85L/100 cases to 0.97L/100 cases, with use still reduced from baseline a year later (1.42L/100 cases) [64].

Education initiatives were also coupled with changes in anesthesia machines or incorporating software to promote low flow rates. Alexander and colleagues looked at the environmental impact of volatile anesthetics after implementation of modern, low-flow anesthetic machines that regulate expired end tidal gas concentrations and adjust flow rates. From 2012 to 2016, they found a difference in CO_2_eq of 8.9 million kg, representing a 66% reduction in GHG emissions and a total volume of volatile anesthetic use from 1703L to 1173L [65]. Similarly, Boyle et al. purchased machines with a lower gas flow rate and educated staff on environmental harm from volatile anesthetics, particularly desflurane. They noted a total volatile spend of €30,943 which equated to a 52% cost saving as well as an 81% reduction in CO_2_eq from 394,126 to 74,004 kg [66]. Carter et al. sought to implement the use of low flow anesthesia and encourage the use of cheaper and environmentally conscious gases like isoflurane. They reported a 25% decrease in total expenditure of volatile agents despite an increase in operating room activity [67]. Glenski and Levine implemented low-flow software on anesthesia machines and education initiatives, achieving 20% decrease in sevoflurane use per month [68].

#### Reuse

Waste: The use of reusable rather than disposable instruments has been well-studied. Gough and colleagues evaluated surgical instrument waste in laparoscopic cholecystectomy, finding that reusable scissors, clip appliers, and ports would save 10.7 kg CO_2_eq per case compared to single-use versions [69]. Similarly, Adler et al. compared waste production from disposable trocars, scissors, and Veress cannula to reusable instruments in laparoscopic cholecystectomy. Single-use instruments generated 1.16 kg household waste, 0.56 kg cardboard waste, and 1.47 kg plastic waste per case [70]. Boberg’s LCA showed a median difference of 446 kg CO_2_eq, 79 potentially disappeared fraction of species in the same area per year (PDF*m^2^*year), 2.4 × 10^–4^ DALYs, and 5160 MJ resource consumption with disposable relative to reusable trocars per laparoscopic cholecystectomy [71]. Similarly, Rizan et al. found environmental superiority of reusable instruments in laparoscopic cholecystectomy [72]. Elsewhere, authors have reported a 56% reduction in expenditure on laparoscopic cholecystectomy using reprocessed trocars, dissectors, curved scissors, jaws, graspers, hooks, and clips [73], as reprocessed devices have not been found to have an increased defect rate compared to original equipment manufacturer devices [74].

Burguburu's LCA identified significant environmental benefits for reusable rather than disposable scrubs, with a 31% lower carbon footprint over a four-year usage period [75]. Reusable surgical gowns offer substantial reductions in natural resource energy consumption (64%), GHG emissions (66%), water usage (83%), and solid waste (84%), assuming a 60-cycle reuse potential [76]. Additionally, reusable surgical basins, gowns, and covers reduced medical waste by 65% in two U.S. medical centers, achieving cost savings of $12.600–$14.000 per hospital [77]. Dettenkofer's comparative analysis between disposable (pulp/PE) and mixed (cotton/synthetic) surgical drapes demonstrated a 5.4 kg lower CO_2_eq, 87.1 MJ lower resource consumption, 72 g lower waste production, and 696 kg lower water consumption associated with the utilization of disposable drapes [78].

Rizan et al. compared carbon and financial costs associated with different modeled scenarios for decontamination and packaging of surgical instruments. Instruments were wrapped individually in flexible pouches, or prepared as surgical sets housed in single-use tray wraps or reusable rigid containers. This resulted in a carbon footprint of 77gCO_2_eq (€1.05) per instrument housed in aluminum containers, 66gCO_2_eq (€1.07) per instrument in tray wrap, and 189gCO_2_eq (€7.35) per individually wrapped instrument. Additionally, incineration of waste increased the carbon footprint of single-use packaging by 33–55%, while this was reduced by up to 10% with appropriate recycling [79]. Other instruments such as the LigaSure have been reprocessed to reduce costs by 55.5% in laparoscopic colectomy [80]. Scissors have been studied, as repairing 17-cm straight Mayo reusable scissors after 40 uses rather than replacing them decreases CO_2_eq emissions by 19% per use and reduces costs from £1.43 to £0.97 [20]. Ibbotson's LCA found that disposable stainless-steel scissors have a 40% higher CO_2_eq impact than plastic versions and a 94% higher impact than reusable stainless-steel scissors, advocating for a 44% cost reduction with reusable options [81]. McGain et al. performed a LCA and found that disposable drug trays in anesthesia cost twice as much, produced 15% more CO_2_ and consumed three times the amount of water compared to reusable trays [82].

#### Recycle

Waste: Amariglio et al. examined elective surgical cases at their institution over one year and identified that 57% of waste was inappropriately discarded, and that 71% of this waste could have been recycled [83]. McKendrick et al. audited the introduction of recycling of paper and cardboard in the theater preparation room and anesthetic room in 20 cases, diverting 54 kg of recycled bags and saving 25 kg CO_2_.[84] Conversely, Wyssusek and colleagues measured the volume of plastic accumulated across 22 ORs, estimating an annual saving of 1,700 kg of recyclable plastic [85].

Efforts have been made to mitigate surgical waste in the OR. In 1992, Tieszen and colleagues surmised that reusable linen products and recycling methods could reduce the weight of surgical waste by 73% [86]. One initiative aimed to reduce plastic waste and recycle theater equipment such as oxygen masks and tubing, advertised the recycling program, and created a dedicated PVC disposal pathway for staff porters over three months. This improved the weight of plastic recycling from operating rooms by 300% [87]. Elsewhere, Martin and colleagues used a systematic approach to improve waste sorting over two weeks, decreasing the weight of solid waste and regulated medical waste by 12% and 59% per OR per day, respectively [88]. Subsequently, Martin et al. used a multi-prong approach to improve waste sorting in the OR and decrease subsequent CO_2_eq emissions over one year. This consisted of educating staff on the importance of sorting infectious versus non-infectious waste, identifying reusable waste, and reorganizing operating rooms to facilitate waste sorting [89].

Sadler et al. introduced reusable metal recycling containers for single-use metal items, collecting 0.14 tons of metal over six weeks, which extrapolated to an annual collection of 1.18 tons [90]. Van Straten et al. examined waste from discarded reusable and disposable stainless-steel instruments in operating rooms across three hospitals by segregating repairable instruments. A total of 1.380 kg of instrument waste was collected, with 237 kg suitable for refurbishment, resulting in cost savings of €38.868 [91, 92]. Dohmen et al. recycled 239 kg of commonly used disposable surgical instruments over six months instead of incinerating them, achieving a reduction of 545 kg CO_2_eq [92]. Rouviere’s LCA targeted sterile medical devices in the OR through 13 actions: seven concerned waste reduction, five focused on waste sorting, and one addressed eco-responsible purchasing of equipment. This reduced 203 tons CO_2_eq emissions and saved 552 m^3^ of water [93].

Anesthesia: McGain et al. conducted a waste audit of OR waste in six operating theaters for five days and found that 66/90 kg of anesthetic waste was non-infectious, and that 58% of this was recyclable [94]. This is higher than a waste audit conducted by Nesaratnam et al. over 1 month with 20 full OR days or 121 cases. Their team found a total of 413 kg of anesthetic waste, of which 136 kg (33%) was recyclable while 277 kg was infectious and or nonrecyclable [95]. Connolly et al. also conducted a waste audit, but with the focus of quantifying volume of glass. Since glass can be recycled, this offers a way to reduce sharps waste which are not recycled into new materials and is more expensive ($0.95/kg sharps vs $0.33/kg glass). Over a one-week period, they found 15.8 kg of glass was able to be recycled [96].

Livingston et al. piloted recycling in several operating rooms by using different bags before patients entered the room and the possibility of contamination occurred. Over one week, this diverted 505.5 kg of waste from the landfill, translating to 25 tons per year. Other researchers have instead focused on reducing infectious regulated medical waste—a more costly and more energy intensive waste stream than general trash [97]. Fraifeld et al. provided education on waste separation in the OR, clarifying which items need to be disposed of via regulated medical waste containers. This decreased the volume of regulated medical waste from 0.33 kg/case to 0.09 kg/case, with a cost savings of $28,392 for their 35 operating rooms over the course of a year [98]. Hubbard et al. discarded anesthetic waste in standard waste bins before patient entry in the operating room, yielding a potential annual reduction of 13,800 kg of regulated medical waste and a cost savings of $2200 in their institution [99].

Education/sustainability programs: There is a growing trend toward implementing team-based strategies to improve quality and sustainability in surgical practice. Wyssusek et al. used a systematic approach to improve waste sorting in the operating room by educating the OR staff and forming a project team with key stakeholders including nursing, anesthesia, OR assistants, directors, and supervisors of perioperative services. The staff was educated regularly, advertisements for appropriate waste sorting were made, waste management change champions were identified to support clinicians in daily practice, and waste segregation and waste recycling programs were implemented over several years. This reduced 60% of unnecessary waste costs, reduced clinical OR waste by 82%, and reduced total OR waste by more than 50% [100]. Wormer and colleagues formed a Green Operating Room Committee including members from corporate leadership, nursing, anesthesia, and OR staff. Their multi-pronged initiative diverted 6.5 tons of medical waste. Recycling all single-use devices reduce annual solid waste by 5,833.2 kg, implementing reusable gel pads rather than disposable OR foam padding saved greater than $50,000 annually, turning off all anesthesia equipment and OR lights when not in use saved $33,000 and 234.3 metric tons of CO_2_ emissions annually, and finally, they showed that converting to alcohol-based scrub would save 2.7 million L of water annually [101].

## Discussion

This scoping review was conducted by the EAES/SAGES joint SSP Taskforce in an effort to establish standard sustainability terminology, consolidate evidence-based outcome measures, define the scope of sustainable action in gastrointestinal surgical practice and assess the impact of prior sustainability initiatives.

**Fig. 5:**
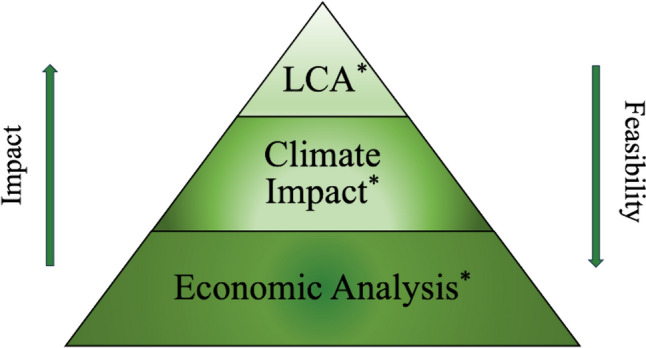
Hierarchy of evidence for sustainability research in surgery

We noted significant heterogeneity in study design and outcome measures across included studies. Most studies were prospective cohort studies while a minority were LCA studies. Yet, LCA studies are the gold standard in sustainability research [102]. While LCA studies provide the most comprehensive review of the overall environmental footprint, they are time and resource-intensive which limits wider use [19]. It may be more feasible to measure climate impact using non-LCA studies with a select but important group of outcomes including resource utilization such as the consumption of energy or water associated with a particular product, process, or service; or via economic analysis of the cost associated with implementing a sustainable initiative or solution (Fig. 5). Non-LCA studies evaluating climate impact or resource use may apply the 10R model of circular economy which provides a structured and practical framework for classifying and measuring the environmental impact of sustainable interventions. The 10R model was endorsed by our SSP Taskforce as a reporting framework for evaluating sustainable initiatives in surgical practice.

Surgical waste production was identified as a major contributor to the environmental footprint of surgical practice. Implementation of a recycling program and education of staff members across operating rooms were among the most impactful interventions in reducing the carbon footprint from surgical practices. The use of leaner equipment kits and reusable surgical instruments also substantially reduced climate impact and associated OR costs. Simple and low-cost initiatives reduce water consumption, including the judicious use of water while scrubbing or the use of alcohol-based scrubs. Importantly, mitigating the use of anesthetic gases, particularly desflurane, also had among the greatest reported improvement in carbon footprint. These represent key areas for institutions to target in their effort to implement sustainability measures in the OR, particularly in gastrointestinal surgery. The importance of establishing a green team with local institutional and division champions was identified as an essential step in supporting the long-term success of a sustainability program [100, 103].

This scoping review has limitations. The broad heterogeneity in study populations, methodology, outcome measures, and data reporting precluded any quantitative synthesis of the comparative effectiveness of interventions on surgical climate impact. Additionally, sustainability expertise from other disciplines such as environmental science and civil engineering were not captured, though these were outside the scope of this review. Some articles are likely not captured despite the comprehensive search applied, due to the variability in aims, methodology, and outcome measures employed across surgical sustainability research. Studies may not have been captured outside of the English, French, German, or Dutch language. Finally, the complex interplay between outcome measures and the permitted degree of flexibility or tailoring of the interventions may limit their evaluation; for instance, the climate impact of equipment used in MIS must be balanced against the indirect positive impact from reduced complications and shorter length of stay. Still, the evidence for sustainability in surgical practice demonstrates the necessity of education, collaboration, and action among the stakeholders in surgical practice.

## Conclusion

This scoping review established standard terminology, outcome measures, and scope across surgical sustainability studies while identifying the most impactful initiatives to reduce the climate impact of surgical practice. The use of standard terminology and SSP outcome measures, will facilitate the interpretation and aggregation of data on the environmental impact of surgical care. Multidisciplinary perioperative team education and collaborations will be required to deploy effective and sustainable initiatives with meaningful impact on reducing the environmental footprint of surgical practice.

## Supplementary Information

Below is the link to the electronic supplementary material.

Supplementary file1 (DOC 37 KB)

Supplementary file2 (DOCX 107 KB)
