# Supplementary material for: Scoping review for the SAGES EAES joint collaborative on sustainability in surgical practice
Source: Surg Endosc. 2024 Aug 22;38(10):5483–504. doi: 10.1007/s00464-024-11141-x (PMC11458728; doi:10.1007/s00464-024-11141-x)
Supplement: Supplementary file 1 — Supplementary file1 (DOC 37 KB) [file 464_2024_11141_MOESM1_ESM.doc]

**Database: OVID Medline Epub Ahead of Print, In-Process & Other Non-Indexed Citations, Ovid MEDLINE(R) Daily and Ovid MEDLINE(R) 1946 to Present (2023 August 25>)**
**Search Strategy:**
**1**  exp Operating Rooms/ (15907)
**2**  (operat* room* or operat* theatre* or operat* theater*).ti,ab,kf. (43109)
**3**  exp General Surgery/ (40584)
**4**  exp Surgical Procedures, Operative/ (3549460)
**5**  (surger* or surgical).ti,ab,kf. (2222837)
**6**  su.fs. (2262375)
**7**  or/1-6 (5056517)
**8**  Environment/ or exp Ecosystem/ (356698)
**9**  exp Carbon Footprint/ (1105)
**10**  (environment or environments or ecoystem* or carbon footprint*).ti,ab,kf. (829979)
**11**  exp Greenhouse Effect/ (6154)
**12**  greenhouse effect*.ti,ab,kf. (851)
**13**  exp Climate Change/ (30323)
**14**  climate chang*.ti,ab,kf. (62335)
**15**  global warm*.ti,ab,kf. (12993)
**16**  "conservation of natural resources"/ or "conservation of energy resources"/ or "conservation of water resources"/ or "environmental restoration and remediation"/ (60620)
**17**  Green Chemistry Technology/ (4644)
**18**  exp Recycling/ (6524)
**19**  recycl*.ti,ab,kf. (68253)
**20**  sustainab*.ti,ab,kf. (138805)
**21**  or/8-20 (1353107)
**22**  exp Electricity/ (99321)
**23**  exp Air Conditioning/ (2882)
**24**  Heating/ (6842)
**25**  (electricit* or air conditioning or heating or cooling or temperatur* conditioning or tempearatur* regulat* or heating).ti,ab,kf. (129232)
**26**  energy efficienc*.ti,ab,kf. (7219)
**27**  energy saving.ti,ab,kf. (3278)
**28**  exp Ventilation/ (6354)
**29**  Lighting/ (12938)
**30**  lighting.ti,ab,kf. (13985)
**31**  exp Waste Management/ (98644)
**32**  (waste adj2 (removal or management or segregation)).ti,ab,kf. (9623)
**33**  (water adj2 (consumption or conservation)).ti,ab,kf. (8074)
**34**  exp Carbon Dioxide/ (99029)
**35**  carbon dioxide.ti,ab,kf. (63686)
**36**  exp Surgical Equipment/ (294867)
**37**  (surgical adj2 (instrument* or gown* or drape* or curtain* or equipment* or stapler*)).ti,ab,kf. (8119)
**38**  Robotic Surgical Procedures/ (16258)
**39**  exp Laparoscopy/ (117957)
**40**  (robotic surgical procedure* or laparoscop*).ti,ab,kf. (154217)
**41**  exp Medical Waste/ (3697)
**42**  (medical waste* or surgical waste*).ti,ab,kf. (1415)
**43**  Disposable Equipment/ (5286)
**44**  (disposable equipment* or single?use* equipment*).ti,ab,kf. (229)
**45**  Equipment Reuse/ (3187)
**46**  ((equipment* or instrument*) adj1 reuse*).ti,ab,kf. (58)
**47**  an?esthetic gas*.ti,ab,kf. (1469)
**48**  Greenhouse Gases/ (2404)
**49**  greenhouse gas*.ti,ab,kf. (14683)
**50**  exp Environmental Pollution/ (631669)
**51**  pollution.ti,ab,kf. (123893)
**52**  or/22-51 (1580605)
**53**  7 and 21 and 52 (5951)

**Database: Embase <1974 to 2023 August 25>**
**Search Strategy:**
**1**  exp operating room/ (51110)
**2**  (operat* room* or operat* theatre* or operat* theater*).ti,ab,kw. (61214)
**3**  exp general surgery/ (20648)
**4**  exp surgery/ (5795402)
**5**  (surger* or surgical).ti,ab,kw. (2904397)
**6**  su.fs. (2368041)
**7**  or/1-6 (6811416)
**8**  environment/ or exp ecosystem/ (688808)
**9**  carbon footprint/ (11196)
**10**  (environment or environments or ecosystem* or carbon footprint*).ti,ab,kw. (1030096)
**11**  greenhouse effect/ or environmental impact/ (49554)
**12**  greenhouse effect*.ti,ab,kw. (981)
**13**  exp climate change/ (59193)
**14**  climate chang*.ti,ab,kw. (60306)
**15**  global warm*.ti,ab,kw. (13748)
**16**  environmental protection/ or energy conservation/ or environmental sustainability/ or green chemistry/ or water conservation/ (76287)
**17**  recycling/ (32639)
**18**  recycl*.ti,ab,kw. (79287)
**19**  sustainab*.ti,ab,kw. (148590)
**20**  or/8-19 (1778268)
**21**  7 and 20 (99974)
**22**  electricity/ (36459)
**23**  air conditioning/ (24782)
**24**  heating/ (41973)
**25**  (electricit* or air conditioning or heating or cooling or temperatur* conditioning or tempearatur* regulat* or heating).ti,ab,kw. (133860)
**26**  energy efficienc*.ti,ab,kw. (7225)
**27**  energy saving*.ti,ab,kw. (4635)
**28**  ventilation.ti,ab,kw. (221020)
**29**  illumination/ (37801)
**30**  lighting.ti,ab,kw. (14369)
**31**  waste management/ or exp solid waste management/ or exp waste disposal/ (69230)
**32**  (waste adj2 (removal or management or segregation)).ti,ab,kw. (12494)
**33**  (water adj2 (consumption or conservation)).ti,ab,kw. (9814)
**34**  carbon dioxide/ (122861)
**35**  carbon dioxide.ti,ab,kw. (65429)
**36**  exp robot assisted surgery/ (26848)
**37**  exp laparoscopy/ (197559)
**38**  (robotic surgical procedure* or robotic assisted surger* or laparoscop*).ti,ab,kw. (251660)
**39**  exp hospital waste/ (4115)
**40**  (medical waste* or surgical waste*).ti,ab,kw. (1778)
**41**  exp disposable equipment/ (10690)
**42**  (disposable equipment* or single-use* equipment*).ti,ab,kw. (427)
**43**  ((equipment* or instrument*) adj1 reuse*).ti,ab,kw. (50)
**44**  anesthetic gas/ (908)
**45**  an?esthetic gas*.ti,ab,kw. (1873)
**46**  greenhouse gas/ (8576)
**47**  greenhouse gas*.ti,ab,kw. (15610)
**48**  exp pollution/ (457103)
**49**  pollution.ti,ab,kw. (142396)
**50**  exp surgical equipment/ (543191)
**51**  (surgical adj2 (instrument* or gown* or drape* or curtain* or equipment* or stapler*)).ti,ab,kw. (8687)
**52**  or/22-51 (1900404)
**53**  21 and 52 (12931)
**54**  exp animals/ not (exp animals/ and exp humans/) (5130342)
**55**  53 not 54 (11909)
**56**  exp animal product/ (299261)
**57**  55 not 56 (11812)
**58**  limit 57 to "preprint (unpublished, non-peer reviewed)" (12)
**59**  57 not 58 (11800)

Scopus: Inception to 2023 August 25

**(operat* room* or operat* theatre* or operat* theater* or surger* or surgical)** (Topic) and **(environment or environments or ecosystem* or carbon footprint* or greenhouse effect* or climate chang* or global warm* or conservation or sustainab* or recycl*)** (Topic) and **(electricit* or air conditioning or heating or cooling or temperatur* conditioning or tempearatur* regulat* or heating or energy efficienc* or energy saving* or ventilation or lighting or waste removal or waste segregation or waste management or water consumption or water conservation or carbon dioxide or robotic surgical procedure* or robotic assisted surger* or laparoscop* or medical waste* or surgical waste* or disposable equipment* or single-use* equipment* or equipment* reuse* or instrument* reuse* or anesthetic gas* or anaesthetic gas* or greenhouse gas* or pollution* or surgical instrument* or surgical gown* or surgical glove* or surgical drape* or surgical mask* or surgical curtain* or surgical equipment* or surgical stapler*)**

- 8028 results
